# Synthesis, anticancer and apoptosis-inducing activities of quinazoline–isatin conjugates: epidermal growth factor receptor-tyrosine kinase assay and molecular docking studies

**DOI:** 10.1080/14756366.2017.1344981

**Published:** 2017-07-18

**Authors:** Adel S. El-Azab, Abdullah Al-Dhfyan, Alaa A.-M. Abdel-Aziz, Laila A. Abou-Zeid, Hamad M. Alkahtani, Abdulrahman M. Al-Obaid, Manal A. Al-Gendy

**Affiliations:** a Department of Pharmaceutical Chemistry, College of Pharmacy, King Saud University, Riyadh, Saudi Arabia;; b Department of Organic Chemistry, Faculty of Pharmacy, Al-Azhar University, Cairo, Egypt;; c Stem Cell & Tissue Reengineering Program, King Faisal Specialized Hospital and Research Center, Riyadh, Saudi Arabia;; d Department of Pharmacology & Toxicology, Collage of Pharmacy, King Saud University, Riyadh, Saudi Arabia;; e Department of Medicinal Chemistry, Faculty of Pharmacy, Mansoura University, Mansoura, Egypt;; f Department of Pharmaceutical Organic Chemistry, Faculty of Pharmacy, Mansoura University, Mansoura, Egypt

**Keywords:** Synthesis, isatin, quinazolinone, antitumor, EGFR-TK, molecular docking

## Abstract

A new series of quinazolinone compounds **16**–**34** incorporating isatin moieties was synthesized. The antitumor efficacy of the compounds against MDA-MB-231, a breast cancer cell line, and LOVO, a colon cancer cell line, was assessed. Compounds **20**, **21**, **22**, **23**, **25, 27**, **28**, **29**, **30**, **31**, **32**, **33**, and **34** displayed potent antitumor activity against MDA-MB-231 and LOVO cells (IC_50_: 10.38–38.67 μM and 9.91–15.77 μM, respectively); the comparative IC_50_ values for 5-fluorouracil and erlotinib in these cells lines were 70.28 μM, 22.24 μM and 15.23 μM, 25.31 μM respectively. The EGFR-TK assay and induction of apoptosis for compound **31** were investigated to assess its potential cytotoxic activity as a representative example of the novel synthesized compounds. At a concentration of 10 μM, compound **31** exhibited efficient inhibitory effect against EGFR-TK and induced apoptosis in MDA-MB-231 cells. Furthermore, a molecular docking study for compound **31** and erlotinib was performed to verify the binding mode toward the EGFR kinase enzyme, and showed a similar interaction as that with erlotinib alone.

**Graphical Abstract:** Compound **31** showed potent antitumor activity and efficient inhibitory effect against EGFR-TK and induced apoptosis of MDA-MB-231 cells at a concentration of 10 μM.

## Graphical Abstract

**Figure:**
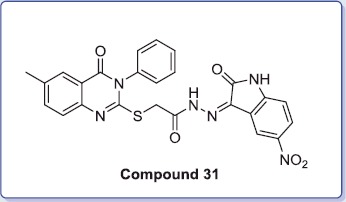

## Introduction

Cancer is one of the most worldwide dangerous health problems and is one of the leading causes of death^1^. Many of the current anticancer agents are highly toxic and nonspecific, so the production of innovative, safe, and selective anticancer molecules is an important goal for the medicinal chemistry researchers. The quinazolinone scaffold is a vital structure in medicinal chemistry^2–22^.

**Figure 1.**
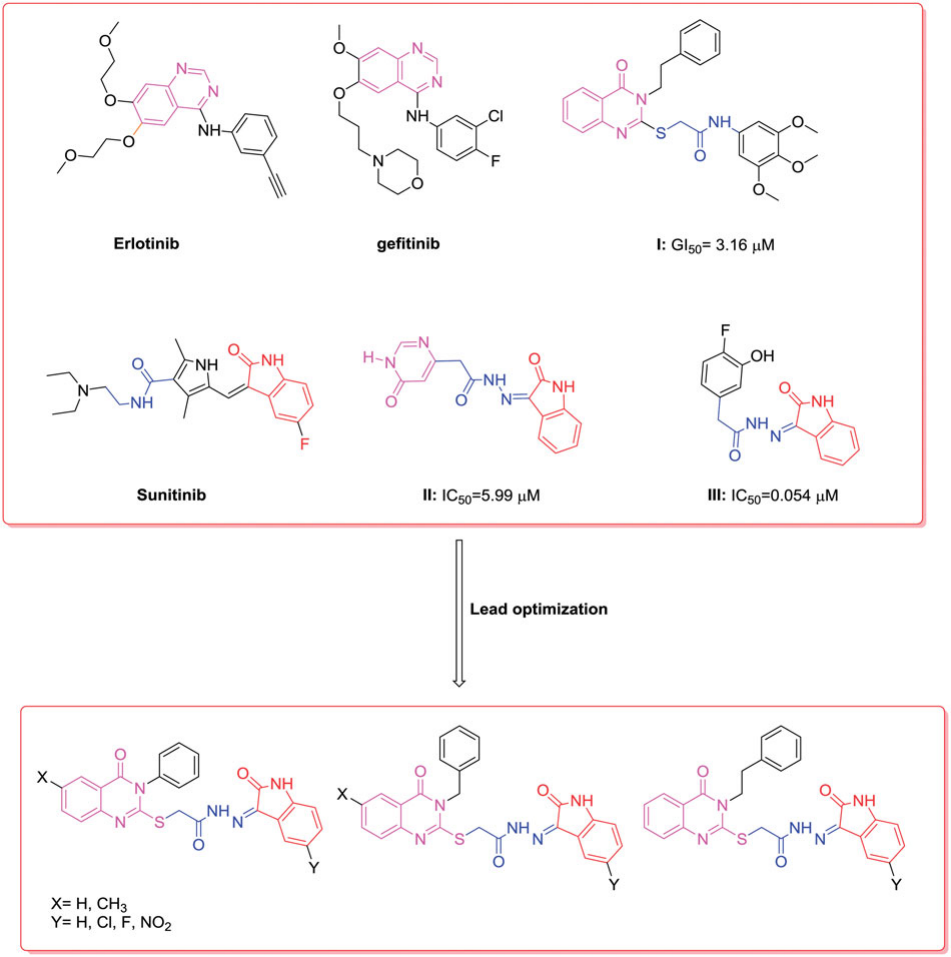
Reported and proposed quinazoline–isatin conjugates with antitumor and tyrosine kinase inhibitory activity.

Anilinoquinazolines, such as gefitinib^23^^,^^24^ and erlotinib^25^, have been established as EGFR kinase inhibitors for the treatment of breast cancer (Figure 1). The 3-phenethylquinazoline derivative (I) has broad spectrum antitumor activity with a mean GI_50_ value of 3.16 μM, in addition to EGFR-TK inhibitory activity^11^ (Figure 1).

Additionally, isatin derivatives exhibit broad spectrum biological effects such as anticancer activity^26^. A 5-fluoro-3-substituted isatin analog (Sunitinib) was approved by the FDA for the treatment of renal carcinoma and gastrointestinal stromal tumors^27^^,^^28^ (Figure 1).

Methyl 3-(1-(4-bromobenzyl)-2,3-dioxoindolin-5-yl)acrylate showed broad spectrum anticancer activity and a weak cytotoxic effect in normal human cells^29^. A series of indolinone hydrazides, including 2-(6-oxo-1,6-dihydropyrimidin-4-yl)-*N′*-(2-oxoindolin-3-ylidene)acetohydrazide (II) and 2-(4-fluoro-3-hydroxyphenyl)-*N′*-(2-oxoindolin-3-ylidene)acetohydrazide (III), were reported as potent anticancer agents with IC_50_ values of 5.99 and 0.054 μM, respectively^30^ (Figure 1). As an attempt to develop effective cytotoxic agents, we synthesized hybrids of quinazoline conjugated to 5-substituted isatin that contained an acylhydrazone moiety and evaluated their cytotoxic activity. Additionally, the EGFR-TK assay and apoptosis induction were investigated for the most active compound, as a representative example of the novel synthesized compounds, to identify their potential cytotoxic activity. A molecular docking study was conducted to verify the structural requirements of the antitumor activity of the target molecules and to support the results of binding of the active compounds to EGFR^31^.

## Materials and methods

### Chemistry

Melting points were recorded on Barnstead 9100 Electrothermal melting point apparatus (UK). IR spectra (KBr) were recorded on a FT-IR Perkin-Elmer spectrometer (Perkin Elmer Inc., MA). Nuclear magnetic resonance (^1^H and ^13^C NMR) spectra were recorded on Bruker 500 or 700 MHz spectrometers (Zurich, Switzerland) using DMSO-d6 as the solvent. Microanalytical data (C, H, and N) were performed on a Perkin-Elmer 240 analyzer (Perkin Elmer Inc., MA) and agreed with the proposed structures within ±0.4% of the theoretical values. Mass spectra were recorded on a Varian TQ 320 GC/MS/MS mass spectrometer (Varian, Palo Alto, CA). 2-[(3-Substituted-4(3H)-quinazolinon-2-yl)thio]acetohydrazides (**11**–**15**) were prepared according to previously reported methods^11^^,^^19^^,^^22^.

#### Synthesis of 2-((3-substituted-4-oxo-3,4-dihydroquinazolin-2-yl)thio)-N'-(2-oxoindolin-3-ylidene)acetohydrazides (16–34)

An equimolar amount of the appropriate 2-[(3-substituted-4(3*H*)-quinazolinon-2-yl)thio]acetohydrazide (**11**–**15**) and substituted isatin was added to methanol (15 ml) containing glacial acetic acid (0.2 ml) and refluxed for 4–6 h. The reaction mixture was filtered while hot; the solid obtained was washed with methanol and dried.

#### 2-((3-Benzyl-4-oxo-3,4-dihydroquinazolin-2-yl)thio)-N'-(2-oxoindolin-3-ylidene)acetohydrazide (16)

Yield: 83%; mp: 250–251 ^o^C; IR (KBr, cm^−1^) *ν*: 3421, 3160 (2NH), 1744, 1725, 1693 (3C=O); ^13^C-NMR (176 MHz, DMSO-d_6_): *δ* 29.5, 47.5, 111.6, 115.6, 119.1, 122.1, 123.1, 126.3, 126.6, 127.1, 127.2, 127.9, 129.0, 135.3, 135.4, 136.0, 142.9, 147.1, 156.9, 161.2, 163.0; ^1^H-NMR (700 MHz, DMSO-d_6_): *δ* 11.54 (s, 0.5H), 11.33 (s, 0.5H), 10.86 (s, 0.5H), 8.14 (s, 0.5H), 8.11 (d, 1H, *J* = 5.5 Hz), 7.75 (s, 1H), 7.57–7.46 (m, 2H), 7.41–7.28 (m, 6H), 7.08–7.03 (m, 1H), 6.93 (d, 1H, *J* = 5.5 Hz), 5.38 (s, 2H), 4.69 (s, 1H), 4.24 (s, 1H); MS: [*m/z*, 469].

#### 2-((3-Benzyl-4-oxo-3,4-dihydroquinazolin-2-yl)thio)-N'-(5-chloro-2-oxoindolin-3-ylidene)acetohydrazide (17)

Yield: 83%; mp: 275–276 ^o^C; IR (KBr, cm^−1^) *ν*: 3448, 3178 (2NH), 1723, 1718, 1695 (3C=O); ^13^C NMR (176 MHz, DMSO-d_6_): *δ* 29.4, 47.5, 113.1, 116.8, 119.1, 120.8, 121.9, 126.3, 126.6, 127.1, 127.2, 127.9, 129.0, 135.3, 135.4, 136.0, 141.6, 147.0, 147.1, 156.8, 161.2; ^1^H-NMR (700 MHz, DMSO-d_6_): *δ* 11.77 (s, 0.5 H), 11.44 (s, 0.5 H), 10.98 (s, 0.5 H), 8.36 (s, 0.5 H), 8.10 (d, 1H, *J* = 6.5 Hz), 7.74 (t, 1H, *J* = 5.5 Hz), 7.65–7.29 (m, 9H), 6.99–6.93 (m, 1H), 5.38 (s, 2H), 4.68 (s, 1H), 4.25 (s, 1H); MS: [*m/z*, 503; M + 2, 505].

#### 2-((3-Benzyl-4-oxo-3,4-dihydroquinazolin-2-yl)thio)-N'-(5-fluoro-2-oxoindolin-3-ylidene)acetohydrazide (18)

Yield: 83%; mp: 244–245 ^o^C; IR (KBr, cm^−1^) *ν*: 3410, 3169 (2NH), 1717, 1702, 1692 (3C=O); ^13^C-NMR (176 MHz, DMSO-d_6_): *δ* 29.3, 47.5, 108.4, 112.7, 118.5, 119.1, 121.5, 126.4, 126.7, 127.1, 127.2, 127.9, 129.0, 134.5, 135.4, 135.9, 139.2, 147.0, 156.2, 158.1, 159.4, 161.2, 163.0; ^1^H-NMR (700 MHz, DMSO-d_6_): *δ* 13.48 (s, 0.4H), 12.74 (s, 0.6H), 11.38 (s, 1H), 8.13 7.36 (m, 12H), 5.40 (s, 2H), 4.70 (s, 1H), 4.28 (s, 1H); MS: [*m/z*, 487].

#### 2-((3-Benzyl-4-oxo-3,4-dihydroquinazolin-2-yl)thio)-N'-(5-nitro-2-oxoindolin-3-ylidene)acetohydrazide (19)

Yield: 83%; mp: 313–315 ^o^C; IR (KBr, cm^−1^) *ν*: 3467, 3279 (2NH), 1741, 1701, 1655 (3C=O); ^13^C-NMR (176 MHz, DMSO-d_6_): *δ* 47.5, 111.2, 111.8, 115.5, 119.1, 121.0, 122.1, 126.3, 126.6, 127.1, 127.2, 127.9, 128.0, 129.0, 135.3, 135.4, 136.0, 142.5, 143.2, 147.0, 148.1, 156.8, 161.2; ^1^H-NMR (700 MHz, DMSO-d_6_): *δ* 12.27 (s, 0.5H), 11.93 (s, 0.5H), 11.56 (s, 0.5H), 9.12 (s, 0.5H), 8.34 (dd, 1H, *J* = 8.5 Hz), 8.10 (d, 1H, *J* = 8.0 Hz), 7.72 (t, 1H, *J* = 7.5 and 8.0 Hz), 7.45 (t, 1H, *J* = 7.5 Hz), 7.37–7.27 (m, 6H), 7.11 (d, 1H, *J* = 9.0 Hz), 5.38 (s, 2H), 4.66 (s, 1H), 4.30 (s, 1H); MS: [*m/z*, 514].

#### 2-((3-Benzyl-6-methyl-4-oxo-3,4-dihydroquinazolin-2-yl)thio)-N'-(2-oxoindolin-3-ylidene)acetohydrazide (20)

Yield: 83%; mp: 270–271 ^o^C; IR (KBr, cm^−1^) *ν*: 3412, 3273 (2NH), 1793, 1724, 1686 (3C=O); ^13^C-NMR (125 MHz, DMSO-d_6_): *δ* 20.7, 39.9, 46.9, 110.6, 115.2, 118.4, 121.6, 125.9, 126.7, 127.4, 128.5, 135.6, 136.0, 144.7, 155.2, 160.7; ^1^H-NMR (500 MHz, DMSO-d_6_): *δ* 11.51–11.32 (m, 1H), 10.84 (d, 1H, *J* = 7.0 Hz), 8.13 (s, 1H), 7.88 (d, 1H, *J* = 4.5 Hz), 7.55–7.32 (m, 8H), 7.06–6.92 (m, 2H), 5.37 (d, 2H, *J* = 10.0 Hz), 4.65 (s, 1H), 4.41 (s, 1H), 2.41 (d, 3H, *J* = 11.0 Hz); MS: [*m/z*, 483].

#### 2-((3-Benzyl-6-methyl-4-oxo-3,4-dihydroquinazolin-2-yl)thio)-N'-(5-chloro-2-oxoindolin-3-ylidene)acetohydrazide (21)

Yield: 83%; mp: 246–247 ^o^C; IR (KBr, cm^−1^) *ν*: 3456, 3163 (2NH), 1741, 1713, 1685 (3C=O); ^13^C-NMR (176 MHz, DMSO-d_6_): *δ* 21.2, 39.6, 47.4, 113.2, 116.8, 126.1, 126.4, 127.2, 127.9, 129.0, 136.1, 136.6, 141.6, 145.2, 155.7, 161.2; ^1^H-NMR (700 MHz, DMSO-d_6_): *δ* 11.74 (s, 0.5H), 11.42 (s, 0.5H), 10.96 (s, 0.5H), 8.36 (s, 0.5H), 7.90 (s, 1H), 7.68–7.56 (m, 2H), 7.50–7.28 (m, 7H), 6.94 (d, 1H, *J* = 5.5 Hz), 5.37 (s, 2H), 4.67 (s, 1.5H), 4.23–4.12 (m, 0.5H), 2.41 (s, 3H); MS: [*m/z*, 517; M + 2, 519].

#### 2-((3-Benzyl-6-methyl-4-oxo-3,4-dihydroquinazolin-2-yl)thio)-N'-(5-fluoro-2-oxoindolin-3-ylidene)acetohydrazide (22)

Yield: 83%; mp: 272–274 ^o^C; IR (KBr, cm^−1^) *ν*: 3448, 3182 (2NH), 1762, 1717, 1686 (3C=O); ^13^C-NMR (125 MHz, DMSO-d_6_): *δ* 20.6, 34.6, 46.9, 111.3, 112.2, 113.2, 113.4, 115.5, 115.6, 118.4, 125.6, 125.8, 126.7, 127.4, 128.5, 135.6, 135.8, 136.0, 138.7, 140.1, 144.7, 155.2, 156.5, 158.4, 160.7, 164.6; ^1^H-NMR (500 MHz, DMSO-d_6_): *δ* 11.58 (s, 0.5H), 11.31 (s, 0.5H), 10.84 (s, 1H), 8.17 (d, 1H, *J* = 8.0 Hz), 7.88 (s, 1H), 7.54 (dd, 1H, *J* = 1.5 and 7.0 Hz), 7.36–7.25 (m, 7H), 6.93–6.90 (m, 1H), 5.37 (s, 2H), 4.65 (s, 1H), 4.59 (s, 1H), 2.39 (s, 3H); MS: [*m/z*, 501].

#### 2-((3-Benzyl-6-methyl-4-oxo-3,4-dihydroquinazolin-2-yl)thio)-N'-(5-nitro-2-oxoindolin-3-ylidene)acetohydrazide (23)

Yield: 83%; mp: 292–294 ^o^C; IR (KBr, cm^−1^) *ν*: 3467, 3167 (2NH), 1741, 1702, 1687 (3C=O); ^13^C-NMR (176 MHz, DMSO-d_6_): *δ* 21.1, 40.4, 47.48, 111.9, 115.5, 116.3, 118.9, 121.0, 122.0, 126.3, 126.4, 127.2, 127.9, 129.0, 136.1, 136.3, 136.6, 136.6, 142.5, 143.2, 145.1, 148.0, 155.7, 161.2, 165.4; ^1^H-NMR (700 MHz, DMSO-d_6_): *δ* 12.56 (0.5H), 11.92 (0.5H), 11.53 (0.5H), 9.12 (0.5H), 8.29 (dd, 1H, *J* = 5.5 and 15.0 Hz), 7.88 (s, 1H), 7.54 (d, 1H, *J* = 5.5 Hz), 7.36–7.27 (m, 7H), 7.10 (d, 1H, *J* = 6.0 Hz), 5.37 (s 2H), 4.60 (s, 1H), 4.27 (s, 1H), 2.40 (s, 3H); MS: [*m/z*, 528].

#### 2-((4-Oxo-3-phenyl-3,4-dihydroquinazolin-2-yl)thio)-N'-(2-oxoindolin-3-ylidene)acetohydrazide (24)

Yield: 83%; mp: 304–305 ^o^C; IR (KBr, cm^−1^) *ν*: 3449, 3223 (2NH), 1726, 1712, 1698 (3C=O); ^13^C-NMR (125 MHz, CDCl_3_-DMSO-d_6_): *δ* 34.6, 111.0, 119.4, 119.6, 120.538, 120.8, 122.4, 126.0, 126.4, 129.3, 129.4, 129.9, 131.5, 134.6, 135.4, 137.6, 142.4, 146.9, 155.7, 160.6, 162.4, 164.9; ^1^H NMR (500 MHz, DMSO-d_6_): *δ* 13.49 (s, 0.56H), 12.72 (s, 0.46H), 11.26 (s, 1H), 8.07 (dd, 1H, *J* = 1.0 and 8.0 Hz), 7.74 (s, 1H), 7.60–7.42 (m, 8H), 7.33 (t, 1H, *J* = 8.0 Hz), 7.0526 (d, 1H, *J* = 6.0 Hz), 6.95–6.87 (m, 1H), 4.55 (s, 1H), 4.08 (s, 1H); MS: [*m/z*, 455].

#### N'-(5-chloro-2-oxoindolin-3-ylidene)-2-((4-oxo-3-phenyl-3,4-dihydroquinazolin-2-yl)thio)acetohydrazide (25)

Yield: 83%; mp: 328–329 ^o^C; IR (KBr, cm^−1^) *ν*: 3447, 3259 (2NH), 1730, 1702, 1659 (3C=O); ^13^C-NMR (176 MHz, DMSO-d_6_): *δ* 21.2, 47.4, 47.4, 113.2, 116.8, 126.1, 126.4, 127.2, 127.9, 129.0, 136.1, 136.6, 141.6, 145.2, 155.7, 161.2; ^1^H-NMR (700 MHz, DMSO-d_6_): *δ* 13.42 (s, 0.5H), 12.63 (s, 0.5H), 11.43 (s, 1H), 8.07 (d, 1H, *J* = 5.5 Hz), 7.79 (s, 1H), 7.72–7.61 (m, 4H), 7.51–7.42 (m, 5H), 6.99–6.93 (m, 1H), 4.57 (s, 1H), 4.12 (s, 1H); MS: [*m/z*, 489; M + 2, 491].

#### N'-(5-fluoro-2-oxoindolin-3-ylidene)-2-((4-oxo-3-phenyl-3,4-dihydroquinazolin-2-yl)thio)acetohydrazide (26)

Yield: 83%; mp: 310–312 ^o^C; IR (KBr, cm^−1^) *ν*: 3429, 3256 (2NH), 1733, 1709, 1686 (3C=O); ^13^C-NMR (125 MHz, DMSO-d_6_): *δ* 34.7, 108.0, 112.2, 118.1, 119.4, 120.9, 126.0, 126.5, 129.4, 129.5, 130.0, 134.8, 135.6, 138.69 146.9, 157.3, 159.2, 160.5, 162.6; ^1^H-NMR (500 MHz, DMSO-d_6_): *δ* 13.47 (s, 0.5H), 12.69 (s, 0.5 H), 11.34 (s, 1H), 8.06 (d, 1H, *J* = 8.0 Hz), 7.77 (s, 1H), 7.61–7.34 (m, 8H), 7.20 (s, 1H) 6.95– 6.90 (m, 1H), 4.56 (s, 1H), 4.11 (s, 1H); MS: [*m/z*, 473].

#### N'-(5-nitro-2-oxoindolin-3-ylidene)-2-((4-oxo-3-phenyl-3,4-dihydroquinazolin-2-yl)thio)acetohydrazide (27)

Yield: 83%; mp: 337–338 ^o^C; IR (KBr, cm^−1^) *ν*: 3431, 3188 (2NH), 1730, 1712, 1691 (3C=O); ^1^H-NMR (500 MHz, DMSO-d_6_): *δ* 13.31 (s, 0.5 H), 12.53 (s, 0.5), 11.94 (s, 1H), 8.29 (d, 2H, *J* = 6.5 Hz), 8.00 (d, 1H, *J* = 7.5 Hz), 7.78 (s, 1H), 7.61–7.47 (m, 8H), 7.13 (s, 1H), 4.61 (s, 1H), 4.16 (s, 1H); MS: [*m/z*, 500].

#### 2-((6-Methyl-4-oxo-3-phenyl-3,4-dihydroquinazolin-2-yl)thio)-N'-(2-oxoindolin-3-ylidene)acetohydrazide (28)

Yield: 83%; mp: 305–306 ^o^C; IR (KBr, cm^−1^) *ν*: 3421, 3298 (2NH), 1725, 1695, 1652 (3C=O); ^13^C-NMR (176 MHz, DMSO-d_6_): *δ* 21.1, 35.1, 111.6, 115.7, 119.7, 120.1, 121.3, 122.1, 123.1, 126.2, 126.3, 129.9, 130.0, 130.4, 136.3, 136.4, 136.5, 136.6, 142.9, 144.3, 145.6, 161.1, 163.0, 165.0; ^1^H-NMR (700 MHz, DMSO-d_6_): *δ* 11.46 (s, 0.5H), 11.31 (s, 0.5H), 10.85 (s, 0.5H), 8.15 (s, 0.5H), 7.86 (s, 1H), 7.63–7.48 (m, 7H), 7.40–7.35 (m, 2H), 7.05 (t, 1H, *J* = 5.0 and 5.5 Hz), 6.97–6.90 (m, 1H), 4.55 (s, 1H), 4.28 (s, 0.5H), 4.08 (s, 0.5 H), 2.40 (s, 3H); MS: [*m/z*, 469].

#### N'-(5-chloro-2-oxoindolin-3-ylidene)-2-((6-methyl-4-oxo-3-phenyl-3,4-dihydroquinazolin-2-yl)thio)acetohydrazide (29)

Yield: 83%; mp: 328–330 ^o^C; IR (KBr, cm^−1^) *ν*: 3419, 3149 (2NH), 1721, 1689, 1646 (3C=O); ^1^H-NMR (500 MHz, DMSO-d_6_): *δ* 11.70 (s, 0.5H), 11.44 (s, 0.5H), 10.95 (s, 1H), 8.35 (s, 1H), 7.87 (s, 1H), 7.60–7.49 (m, 7H), 7.36 (s, 1H), 6.93 (d, 1H, *J* = 8.0 Hz), 4.51 (s, 1H), 4.35 (s, 0.75H), 4.10 (s, 0.25H), 2.42 (s, 3H); MS: [*m/z*, 503; M + 2, 505].

#### N'-(5-fluoro-2-oxoindolin-3-ylidene)-2-((6-methyl-4-oxo-3-phenyl-3,4-dihydroquinazolin-2-yl)thio)acetohydrazide (30)

Yield: 83%; mp: 281–282 ^o^C; IR (KBr, cm^−1^) *ν*: 3448, 3283 (2NH), 1725, 1699, 1662 (3C=O); ^13^C-NMR (125 MHz, DMSO-d_6_): *δ* 20.6, 34.6, 111.3, 112.2, 119.2, 121.0, 125.7, 125.8, 129.4, 129.5, 129.9, 135.6, 135.8, 136.0, 136.1, 138.6, 145.0, 145.1, 155.6, 156.5, 157.3, 159.2, 160.5, 162.6, 164.6; ^1^H-NMR (500 MHz, DMSO-d_6_): *δ* 11.57 (s, 0.4H), 11.33 (s, 0.6H), 10.83 (s, 0.4H), 8.14 (s, 0.6H), 7.86 (s, 1H), 7.61–7.49 (m, 7H), 7.35 (s, 1H), 7.26–7.21 (m, 1H), 6.91 (t, 1H, *J* = 4.0 Hz), 4.55 (s, 1.4H), 4.09 (s, 0.6H), 2.41 (s, 3H); MS: [*m/z*, 487].

#### 2-((6-Methyl-4-oxo-3-phenyl-3,4-dihydroquinazolin-2-yl)thio)-N'-(5-nitro-2-oxoindolin-3-ylidene)acetohydrazide (31)

Yield: 83%; mp: 344–345 ^o^C; IR (KBr, cm^−1^) *ν*: 3446, 3196 (2NH), 1744, 1707, 1648 (3C=O); ^1^H-NMR (700 MHz, DMSO-d_6_): *δ* 13.29 (s, 0.7H), 12.52 (s, 0.3H), 11.92 (s, 0.7H), 11.52 (s, 0.3H), 8.30 (s, 1H), 7.87 (s, 1H), 7.60–7.11 (m, 9H), 4.59 (s, 1H), 4.35 (s, 0.3H), 4.14 (s, 0.7H), 2.41 (s, 1H); MS: [*m/z*, 514].

#### 2-((4-Oxo-3-phenethyl-3,4-dihydroquinazolin-2-yl)thio)-N'-(2-oxoindolin-3-ylidene)acetohydrazide (32)

Yield: 83%; mp: 273–274 ^o^C; IR (KBr, cm^−1^) *ν*: 3448, 3133 (2NH), 1715, 1686, 1636 (3C=O); ^13^C-NMR (176 MHz, DMSO-d_6_): *δ* 33.9, 40.4, 46.0, 111.1, 111.5, 119.2, 120.2, 122.1, 123.1, 126.2, 126.5, 126.9, 127.2, 129.1, 135.1, 135.2, 138.1, 142.9, 147.0, 147.0, 160.8, 160.8; ^1^H-NMR (700 MHz, DMSO-d_6_): *δ* 11.55 (s, 0.5H), 11.28, (s, 0.5H), 10.83 (s, 0.5H), 8.16 (s, 0.5H), 8.15 (d, 1H, *J* = 2.0 Hz), 8.07 (d, 1H, *J* = 5.5 Hz), 7.80 (d, 1H, *J* = 6.0 Hz), 7.68–7.26 (m, 8H), 7.10–7.00 (m, 1H), 6.93–6.90 (m, 1H), 4.74–4.49 (m, 1.5H), 4.41–4.36 (m, 2.5H, *J* = 5.0 and 7.5 Hz), 3.07–3.00 (m, 2H); MS: [*m/z*, 483].

#### N'-(5-chloro-2-oxoindolin-3-ylidene)-2-((4-oxo-3-phenethyl-3,4-dihydroquinazolin-2-yl)thio)acetohydrazide (33)

Yield: 83%; mp: 233–235 ^o^C; IR (KBr, cm^−1^) *ν*: 3469, 3167 (2NH), 1710, 1676, 1646 (3C=O); ^13^C-NMR (176 MHz, DMSO-d_6_): *δ* 33.9, 40.4, 46.0, 112.4, 116.8, 119.2, 121.9, 126.1, 126.2, 126.5, 126.9, 127.2, 129.1, 135.1, 135.2, 138.1, 143.0, 147.0, 156.2, 160.8, 164.8, 172.4; ^1^H-NMR (700 MHz, DMSO-d_6_): *δ* 11.67 (s, 0.5H), 11.58 (s, 0.5H), 10.97 (s, 1H), 8.37 (s, 1H), 8.06 (d, 1H, *J* = 5.5 Hz), 7.70 (t, 1H, *J* = 5.0 Hz), 7.44–7.21 (m, 8H), 6.93 (d, 1H, *J* = 6.0 Hz), 4.70 (s, 1H), 4.52 (s, 1H), 4.29 (t, 2H, *J* = 5.5 Hz), 3.05 (t, 2H, *J* = 5.5 and 5.5 Hz); MS: [*m/z*, 517; M + 2, 519].

#### N'-(5-fluoro-2-oxoindolin-3-ylidene)-2-((4-oxo-3-phenethyl-3,4-dihydroquinazolin-2-yl)thio)acetohydrazide (34)

Yield: 83%; mp: 257–258 ^o^C; IR (KBr, cm^−1^) *ν*: 3442, 3267 (2NH), 1719, 1683, 1639 (3C=O); ^1^H-NMR (500 MHz, DMSO-d_6_): *δ* 11.61 (s, 0.5H), 11.30 (s, 0.5H), 10.85 (s, 0.5H), 8.19 (d, 0.5H, *J* = 8.5 Hz), 8.05 (d, 1H, *J* = 8.0 Hz), 7.70 (t, 1H, *J* = 7.0 and 7.5 Hz), 7.50–7.21 (m, 9H), 6.91 (dd, 1H, *J* = 4.0 and 4.5 Hz), 4.73 (s, 1H), 4.65 (s, 1H), 4.28 (dd, 2H, *J* = 4.0 Hz), 3.05 (t, 2H, *J* = 7.5 Hz); MS: [*m/z*, 501].

## Biology

### WST-1 cell proliferation assay

The cell proliferation assay was conducted according to a previously reported method^32^.

#### Immunofluorescence microscopy

The EGFR immunofluorescence assay was conducted according to a previously reported method^33^.

### Apoptosis assay

Vybrant apoptosis assay kit (Annexin-V, APC conjugate; Molecular Probes™) was used to evaluate cell viability in accordance with the manufacturer’s recommendation^33^.

## Docking methodology

All modeling experiments were conducted with MOE programs running on a PC^34^. Hydrogen bonds with a bond length of up to 3.5 Å were considered. The starting coordinates of the X-ray crystal structure of the EGFR enzyme in complex with erlotinib (PDB code: 1M17) were obtained from the RCSB Protein Data Bank of Brookhaven National Laboratory^35^. All hydrogens were added and the enzyme structure was subjected to a refinement protocol in which the constraints on the enzyme were gradually removed and minimized until the RMS gradient was 0.01 kcal/mol Å. The energy minimization was conducted using the AMBER molecular mechanics force field. The lowest energy conformer, the “global-minima,” was pre-positioned using the crystal structure ligand “erlotinib” as a template at the enzyme-binding pocket.

## Results and discussion

### Chemistry

**Scheme 1.**
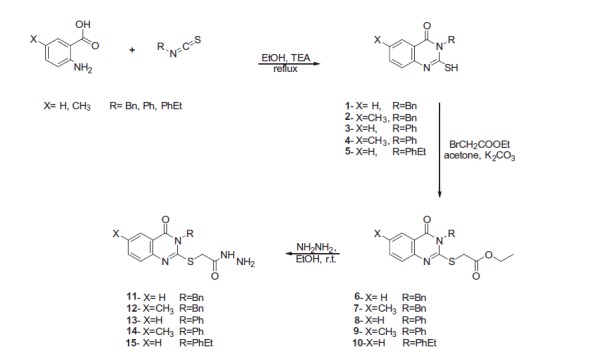
Synthesis of 2-[(3-substituted-4(3*H*)-quinazolinon-2-yl)thio]acetohydrazides **11**–**15**.

2-Mercapto-3-substituted-4(3*H*)-quinazolinones (**1**–**5**) were prepared by heating anthranilic acid derivatives with an appropriate isothiocyanate in ethanol containing a catalytic amount of triethylamine. Accordingly, 2-[(3-substituted-4(3*H*)-quinazolinon-2-yl)thio]acetohydrazides (**11**–**15**) were obtained by stirring compounds **1**–**5** with ethyl 2-bromoacetate in acetone to yield the corresponding ethyl 2-[(3-substituted-4(3*H*)-quinazolinon-2-yl)thio]acetates (**6**–**10**), which were then stirred with hydrazine hydrate in ethanol^11^^,^^19^^,^^22^ (Scheme 1).

**Scheme 2.**
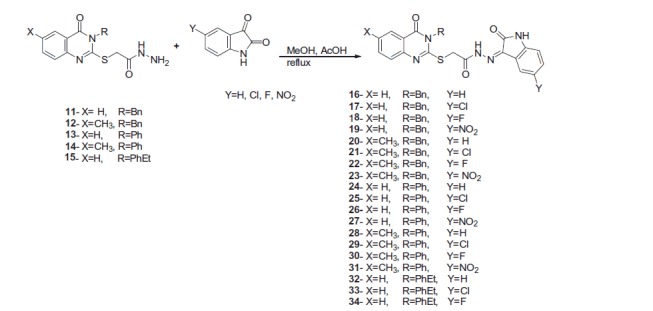
Synthesis of quinazoline-isatin conjugates **16**–**34**.

The 2-[(3-substituted-4-quinazolinon-2-yl)thio]-*N*'-(2-oxoindolin-3-ylidene)acetohydrazides (**16**–**34**) were produced at 80–85% yield by heating an appropriate 2-[(3-substituted-4(3*H*)-quinazolinon-2-yl)thio]acetohydrazide (**11**–**15**) and isatin derivative in methanol containing a catalytic amount of acetic acid^26^ (Scheme 2).

^1^H-NMR of compounds **16**–**34** revealed singlet signals corresponding to the two NH groups at 13.48–10.85 and 11.94–8.13 ppm, in addition to presence of signals for SCH_2_CO at 4.79–4.05 ppm as a mixture of the E/Z isomers. Additionally, the IR spectra of compounds **16**–**34** showed new bands at 3467–3410 cm^–1^ and 3298–3133 cm^–1^, which corresponded to the NH group of amides, and 1793–1713 cm^–1^ and 1676–1725 cm^–1^, owing to the presence of two C=O groups in addition to the C=O of the 4-quinazolinone nucleus at 1698–1636 cm^–1^.

## Biological activity

### Cell proliferation inhibition assay

The *in vitro* antitumor activity of compounds **16**–**34** against the human breast cancer cell line, MDA-MB-231, and the colon cancer cell line, LOVO, was determined by WST-1 assay^32^ using 5-FU and erlotinib as a reference drugs, and IC_50_ was calculated for each cell line (Table 1). In the present study, the active compounds exhibited a characteristic selectivity potential in addition to broad-spectrum antitumor activity.

**Table 1.** *In vitro* antitumor activity of the newly synthesized compounds **16**–**34**.

Compounds	MDA-MB-231^a^ IC_50_ (μM)^c^	LOVO^b^ IC_50_ (μM)^c^
**16**	16.23 ± 0.32	33.97 ± 0.26
**17**	14.97 ± 0.37	23.98 ± 0.06
**18**	12.38 ± 0.3	21.46 ± 0.13
**19**	12.31 ± 0.11	17.53 ± 0.04
**20**	16.82 ± 0.13	14.80 ± 0.1
**21**	14.48 ± 0.03	14.21 ± 0.06
**22**	18.33 ± 0.01	14.14 ± 0.06
**23**	17.14 ± 0.01	13.39 ± 0.23
**24**	11.50 ± 0.36	20.39 ± 0.02
**25**	11.41 ± 0.07	12.00 ± 0.05
**26**	11.80 ± 0.02	23.62 ± 0.01
**27**	18.05 ± 0.04	12.80 ± 0.03
**28**	37.41 ± 0.06	14.20 ± 0.09
**29**	38.67 ± 0.04	14.00 ± 1.02
**30**	13.77 ± 0.4	14.12 ± 0.06
**31**	10.38 ± 0.22	9.91 ± 0.12
**32**	18.35 ± 0.14	16.51 ± 0.15
**33**	20.21 ± 0.05	14.37 ± 0.46
**34**	20.06 ± 0.11	15.77 ± 0.16
**5-FU**	70.28 ± 0.2	15.23 ± 0.09
**Erlotinib**	22.24 ± 0.22	25.31 ± 0.12

a Aggressive human MDA-MB-231 (representative triple negative breast cancer cells with high metastasis potential).

b Aggressive human LOVO colon cell line (type IV metastasized colon cancer).

c IC_50_: concentration of the compound (μM) that produced 50% inhibition of cell growth inhibition after 48 h of treatment.

For the selectivity against the MDA-MB-231 cell line, compounds **16**, **17**, **18**, **19**, **20**, **21**, **22**, **23**, **24**, **25**, **26**, **27**, **30**, **31**, **32**, **33**, and **34** showed high activity (IC_50_: 10.38–20.21 μM); the comparative IC_50_ values for 5-FU and erlotinib were 70.28 and 22.24 μM respectively. On the other hand, compounds **28** and **29** (IC_50_: 37.41 and 38.67 μM); were less active than erlotinib but more active than 5-FU.

Moreover, the LOVO cell line was sensitive toward compounds **19**, **20**, **21**, **22**, **23**, **25**, **27**, **28**, **29**, **30**, **31**, **32**, **33**, and **34** (IC_50_: 9.91–17.53 μM); the comparative IC_50_ value for 5-FU and erlotinib were 15.23 and 25.31 μM respectively. Compounds **17**, **18**, **24**, and **26** were less active than 5-FU with IC_50_ values of 20.39–23.98 μM but more active than erlotinib.

With regards to broad-spectrum antitumor activity, compounds **20**, **21**, **22**, **23**, **25**, **27**, **30**, **31**, **32**, **33**, and **34** showed strong antitumor activities against MDA-MB-231 cells and LOVO cells, which was supported by the IC_50_ values (10.38–20.21 μM and 9.91–15.77 μM, respectively). Moreover, compound **31** showed the highest potency toward MDA-MB-231 cells and LOVO cells with IC_50_ values of 10.38 and 9.91 μM, respectively.

### GFR tyrosine kinase enzyme inhibition assay

**Figure 2.**
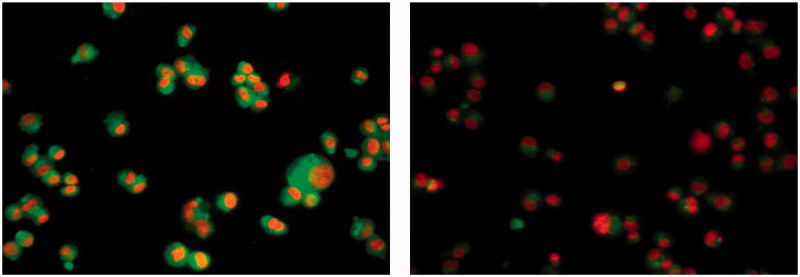
EGFR (left panel; green color) of MDA-MB-231 breast cell line and (right panel) MDA-MB-231 breast cell line after treatment with compound **31**.

The enzyme activity assay of the most active compound **31** toward the MDA-MB-231 breast cancer cell line was selected as representative example of the compounds and administered at a single concentration (10 μM) against EGFR-TK to investigate the mechanism of action of the newly synthesized compounds^33^. The immunofluorescence staining of EGFR in MDA-MB-231 cells treated with compound **31** at 10 μM indicated a good selectivity of compound **31** to EGFR-TK, as shown by inhibition of the level of EGFR on the cell membrane as well as in the nucleus (Figure 2).

### Apoptosis detection by flow cytometry

**Figure 3.**
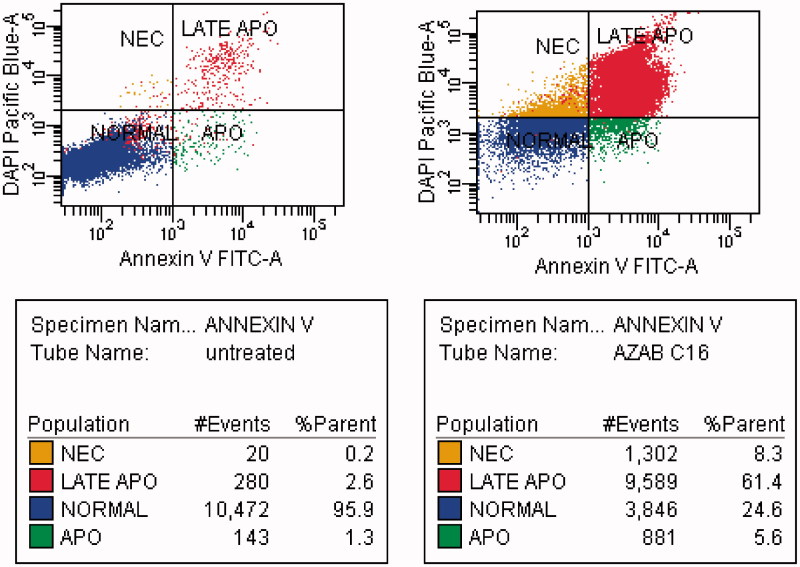
MDA-MB-231 breast cancer cell line was treated with compound **31** (right panel), which displayed an increased percentage of fluorescein isothiocyanate annexin V (Annexin V–FITC), and untreated control cells (left panel).

The effect of compound **31** on the apoptosis was investigated using DAPI (4,6-diamidino-2-phenylindole) and annexin V-FITC biparametric cytofluorimetric analysis^32^. After treatment with compound **31** (10 μM for 24 h), the MDA-MB-231 breast cancer cells were stained with DAPI and annexin V, and analyzed by flow cytometry (Figure 3). Compound **31** was able to induce apoptosis in MDA-MB-231 cells. Compound **31** induced apoptosis by a 30-fold increase in the percentage of fluorescein isothiocyanate annexin V (Annexin V-FITC)-positive apoptotic cells (right panel) in comparison with untreated cells (left panel). Compound **31** increased the percentage of apoptotic cells by 5.6% and late apoptotic cells by 61.4% compared with 1.3% and 2.6% in untreated control cells, respectively. Moreover, the tested compound induced necrosis in treated cells by 8.3% compared with 0.2% in untreated control cells.

### Structure–activity relationships

The structure–activity relationships of the tested compounds revealed that 5-methyl-3-benzyl derivatives **20**–**23** (IC_50_: 14.48–18.33 μM and 13.39–14.80 μM) and 5-methyl-3-phenyl derivatives **28**–**31** (IC_50_: 10.38–38.67 μM and 9.91–14.20 μM) showed significant inhibition of MDA-MB-231 cells and LOVO cells, compared with 5-FU (IC_50_: 70.28 μM and 15.23 μM), respectively (Table 1).

Moreover, unsubstituted 3-benzyl derivatives **16**–**19** (IC_50_: 12.31–16.23 μM and 17.53–33.97 μM), 3-unsubstituted phenyl derivatives **24**–**27** (IC_50_: 11.41–18.05 and 12.0–23.62 μM) and unsubstituted 3-phenethyl derivatives **32**–**34** (IC_50_: 18.35–20.21 and 14.37–17.87 μM) were more selective for MDA-MB-231 cells than LOVO colon cells, compared with 5-FU (IC_50_: 70.28 μM and 15.23 μM), respectively (Table 1).

In MDA-MB-231 cells, the unsubstituted 3-benzyl derivatives **16**–**19** (IC_50_: 12.31–16.23 μM) and unsubstituted 3-phenyl derivatives **24**–**27** (IC_50_: 11.41–18.05 μM) were more active than the 5-methyl-3-benzyl derivatives **20**–**23** (IC_50_: 14.48–18.33 μM) and 5-methyl-3-phenyl derivatives **28**–**31** (IC_50_: 10.38–38.67 μM) respectively. In the LOVO cells, the 5-methyl-3-benzyl derivatives **20**–**23** (IC_50_: 13.39–14.80 μM) and 5-methyl-3-phenyl derivatives **28**–**31** (IC_50_: 9.91–14.2 μM) were more active than the unsubstituted 3-benzyl derivatives **16**–**19** (IC_50_: 17.53–33.97 μM) and unsubstituted 3-phenyl derivatives **24**–**27** (IC_50_: 12.0–23.62 μM), respectively (Table 1).

### Molecular docking results

The antitumor activities of the weakly active compound **28** and the highly active compound **31** in MDA-MB-23 cells, which highly express epidermal growth factor receptor (EGFR)^7^^,^^10^^,^^11^^,^^15^^,^^19^^,^^22^ and the binding activity of compound **31** with EGFR, encouraged us to conduct molecular docking simulations of the binding site of the EGFR kinase.

**Figure 4.**
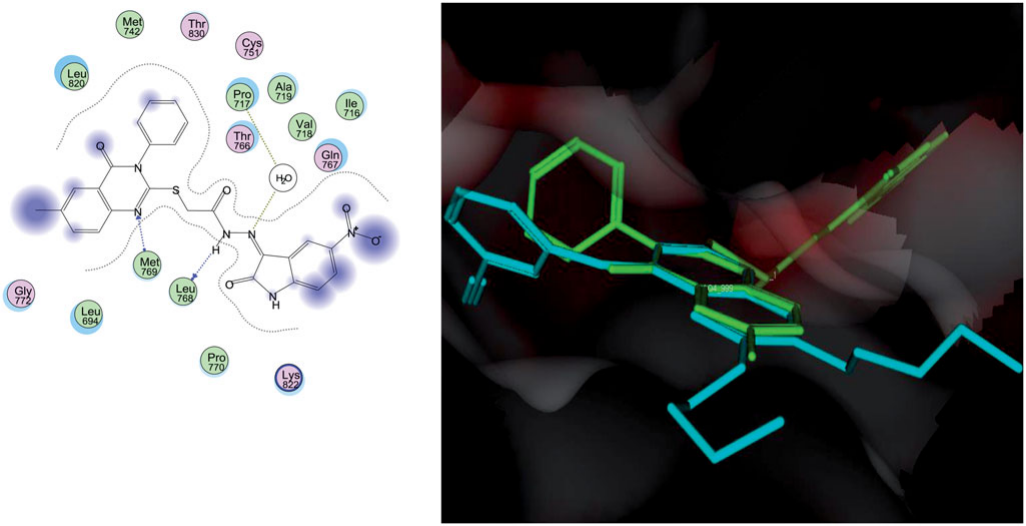
Docking of compound **31** (left panel) and superposition with erlotinib (right panel) in the receptor pocket of EGFR kinase. Compound **31** and erlotinib are shown in green and cyan, respectively.

Compounds **28** and **31** were docked into the receptor active site of EGFR along with their inhibitor erlotinib (Tarceva™) (PDB code: 1M17)^35^. All calculations were performed using MOE 2008.10 software^34^. The docking study of the most active compound **31** revealed that the quinazoline ring typically overlaid the corresponding ring of erlotinib without clashing with the surrounding amino acids. The substituted linkage at the C-2 hybrid of the binding of compound **31** in both the activation and catalytic loops where N1 was uniquely bound with the distinctive residue Met^769^. A semicarbazide nitrogen atoms was recognized via hydrogen bonding with Leu^768^, while the second semicarbazide nitrogen atom performed hydrophilic interaction by cross interaction with Pro^717^ through the water molecule in the pocket. The two adjacent conserved amino acids Leu^768^ and Met^769^ firmly held the backbone of compound **31**, which augmented the recognition and the overall inhibition activity (Figure 4).

**Figure 5.**
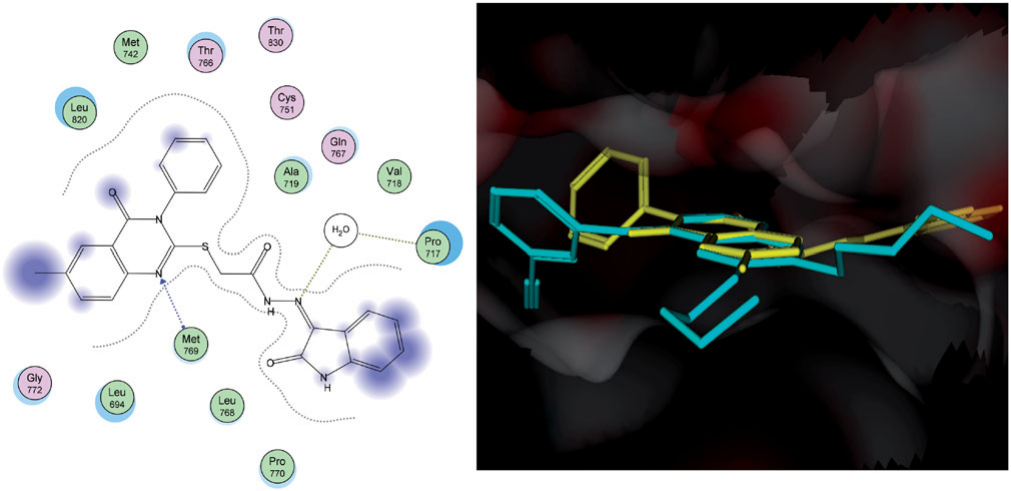
Docking of compound **28** (left panel) and superposition with erlotinib (right panel) in the receptor pocket of EGFR kinase. Compound **28** and erlotinib are shown in yellow and cyan, respectively.

In contrast, compound **28** was bound in different manner, which dramatically lowered the overall complementarity. Although N1 was clearly recognized with hydrogen bonding to the distinctive residue Met^769^, N3 was buried away from the surrounding amino acids owing to the rigidity of the connected phenyl group. However, the semicarbazide linkage enriched the hydrophilic interaction by cross interaction with Pro^717^ through the water molecule in the pocket (Figure 5).

## Conclusions

A new series of quinazolinone-isatin conjugates **16**–**34**, which strongly inhibited growth in the MDA-MB-231 breast cancer cell line and LOVO colon cancer cell line, was synthesized. Compounds **16**–**34** showed high activity against the human MDA-MB-231 breast cell line (IC_50_: 10.38–38.67 μM) in comparison with 5-FU and erlotinib (IC_50_: 70.28 μM and 22.24 μM, respectively). Similarly, compounds **19**–**23**, **25**, and **27**–**34** possessed strong activity against the LOVO colon cancer cell line (IC_50_: 9.91–17.87 μM) in comparison with 5-FU and erlotinib (IC_50_: 15.23 μM and 25.31 μM, respectively). Compounds **20**–**23**, **25**, and **27**–**34** showed potent antitumor activity against the MDA-MB-231 and LOVO cell lines (IC_50_: 10.38–38.67 μM and 9.91–15.77 μM, respectively. Compound **31** inhibited the level of EGFR-TK in the cell membrane, as well as in the nucleus, of MDA-MB-231 cells as a representative example of quinazolinone–isatin conjugates at a single concentration (10 μM). Compound **31** increased the number of apoptotic cells by 5.6% and late apoptotic cells by 61.4% compared with 1.3 and 2.6%, respectively, in untreated control cells. Additionally, compound **31** induced necrosis in treated cells by 8.3% compared with 0.2% in untreated control cells. A molecular docking simulation was performed for compounds **31** and **28** into the binding site of EGFR kinase, which showed a similar binding mode to erlotinib. The results of molecular docking can help in the design of new molecules with potential antitumor activity and good binding to the enzyme receptor site.

## Disclosure statement

No potential conflict of interest was reported by the authors.
